# Evaluation of perinatal anxiety assessment measures: a cognitive interview study

**DOI:** 10.1186/s12884-024-06641-6

**Published:** 2024-07-27

**Authors:** Rose Meades, Andrea Sinesi, Louise R. Williams, Amy Delicate, Helen Cheyne, Margaret Maxwell, Fiona Alderdice, Julie Jomeen, Judy Shakespeare, Cassandra Yuill, Susan Ayers, the MAP Study Team, Catherine Best, Agnes Hann, Debra Salmon, Nazihah Uddin, James Walker, Simon Gilbody

**Affiliations:** 1 Centre for Maternal and Child Health Research, School of Health and Psychological Sciences, City, University of London, Northampton Square, London, EC1V 0HB UK; 2 Nursing, Midwifery and Allied Health Professions Research Unit, Pathfoot Building, University of Stirling, Stirling, FK9 4LA UK; 3 Nuffield Department of Population Health, University of Oxford, Old Road Campus, Oxford, OX3 7LF UK; 4 Southern Cross University, Gold Coast Airport, Terminal Dr, QLD 4225 Bilinga, Australia; 5 Independent Researcher - Retired General Practitioner, Oxford, UK

**Keywords:** Pregnancy, Postpartum, Anxiety, Screening, Assessment

## Abstract

**Background:**

Anxiety in pregnancy and postpartum is highly prevalent but under-recognised. To identify perinatal anxiety, assessment tools must be acceptable, relevant, and easy to use for women in the perinatal period.

**Methods:**

To determine the acceptability and ease of use of anxiety measures to pregnant or postpartum women (*n* = 41) we examined five versions of four measures: the Generalised Anxiety Disorder scale (GAD) 2-item and 7-item versions; Whooley questions; Clinical Outcomes in Routine Evaluation (CORE-10); and Stirling Antenatal Anxiety Scale (SAAS). Cognitive interviews were used to examine ease of comprehension, judgement, retrieval and responding.

**Results:**

All measures were acceptable. Some items were deemed less relevant to the perinatal period e.g., difficulties sleeping. Ease of comprehension, judgement, retrieval and responding varied, with all measures having strengths and weaknesses. The SAAS and CORE-10 had the lowest mean number of problematic components. The GAD had the highest mean number of problematic components​. Non-binary response options were preferred. Preferences for time frames (e.g. one week, one month) varied. Qualitative data provides in-depth information on responses to each measure.

**Conclusions:**

Findings can be used to inform clinical guidelines and research on acceptable anxiety assessment in pregnancy and after birth.

## Background

Anxiety symptoms and disorders are commonly experienced in pregnancy and after birth and occurs in around 20% of women [1]. Problematic anxiety can have a substantial impact on women and infants. This includes increased risk of preterm birth, postnatal depression, and poorer developmental outcomes for the infant. [2, 3] Evidence also suggests anxiety symptoms which do not meet diagnostic thresholds can still be distressing and debilitating for women [4]. Anxiety is highly comorbid with depression [5] and the cost of perinatal mental health problems to society is high – with estimates in the UK of £8.1 billion for every annual cohort of women who give birth [6].

Screening and assessment of anxiety in pregnancy and after birth is therefore important so healthcare professionals (HCPs) can identify women experiencing problematic anxiety and to inform onward clinical assessments, referrals and treatment. This in turn could lead to improved outcomes for women and infants [7]. However, in order to be successful, screening and assessment tools need to be acceptable to perinatal women and effective at identifying those who require support and treatment.

The development of assessment tools historically has not focused on a collaborative approach with perinatal women. Evaluating acceptability is particularly important given the now recognised importance of collaborative approaches in research, including public and participant involvement in research, co-production and the development of patient-derived outcome measures. Perinatal women, who are the lay experts in whether the tool captures all relevant aspects of perinatal anxiety, and whether the tool is acceptable or not, need to have a voice in whether tools used *with* them are relevant *to* them, as per best practice guidance in the development of outcome measures [8].

Clinical guidelines currently recommend routine assessment of perinatal anxiety which were developed in non-perinatal populations. In the UK, the National Institute for Health and Care Excellence (NICE) [9] recommends women are asked two questions to identify anxiety (Generalised Anxiety Disorder scale (GAD-2)) [10] and two questions to identify depression (Whooley questions) [11] during routine maternity care appointments. If women screen positive for anxiety or depression on these questions, it is recommended they are followed-up with longer versions of the measures (the GAD-7 or PHQ-9 respectively) [8]. However, evidence for the acceptability and effectiveness of these measures is limited, [12] with some evidence the GAD may generate many false positives [13].

Another approach to assessment is to use a general mental health questionnaire rather than multiple different tools to assess anxiety or depression. An example of this is the Clinical Outcomes in Routine Evaluation-10 (CORE-10) scale, which is commonly used in psychological services in the UK. The CORE-10 asks about a range of symptoms [14] and appears to have good psychometric properties when used with perinatal women [15], Alternatively, some argue it is important to use measures that include questions about anxiety about pregnancy, birth, or the infant, as this is particularly pertinent perinatally and may predict poor birth outcomes [2]. The Stirling Antenatal Anxiety Scale (SAAS) is an example of a measure that includes both general and pregnancy-specific anxiety symptoms, such as worries about the baby, birth and parenting [16].

For any assessment to be successful the measures need to be acceptable to women and easy to use. Acceptability has been defined as ‘determining how well an intervention [i.e. assessment of anxiety] will be received by the target population and the extent to which the new intervention or its components might meet the needs of the target population’ [17]. One way in which acceptability and ease of use can be examined is through cognitive interviews, which use think aloud techniques and probes to determine ease of comprehension, judgement of questions and responses, ease of retrieving information to answer the question and ease of response to the question [18].

Whilst there are many studies on the psychometric properties of questionnaire measures of mental health during the perinatal period, there is little research reporting the qualitative components of such questionnaires, such as their content validity, acceptability and ease of use. There is some evidence on the acceptability of the Whooley questions [19] but no or little evidence on the acceptability of the GAD-2, GAD-7, CORE-10 or SAAS to women in the perinatal period. This study therefore aims to determine the acceptability and ease of use of these measures to pregnant and postpartum women using cognitive interviews. Measures were chosen based on clinical guidelines, [9] use in perinatal and other clinical populations, clinical utility, relevance to the perinatal period, and psychometric evidence. Five versions of four self-report measures of mental health were tested: the GAD-2, GAD-7, Whooley questions, CORE-10 and SAAS.

## Methods

We used a cognitive interview design based on an established model of cognitive survey responses [18] to determine ease of use and acceptability of questionnaires to assess perinatal anxiety and mental health. We report elsewhere a qualitative interview of women’s experiences and views of perinatal mental health assessment [20].

### Study sample

Women were eligible if they were pregnant or up to six weeks postpartum, aged 16 or over, with sufficient English language to take part in an interview. Potential participants were recruited through UK organisations such as the NCT and Maternal Mental Health Change Agents Scotland. Recruitment methods included social media (e.g. Facebook), attendance at antenatal groups, and word of mouth. Participants were sampled according to pregnancy gestation at 12 weeks (Mean 12·2, SD 1·2), 22 weeks (Mean 21·6, SD 2·7), 31 weeks (Mean 30·4, SD 2·7) and 6 weeks postpartum (Mean 6·1, SD 1·9) in two nations (England/Scotland). We sampled women who scored above and below thresholds on measures recommended by NICE clinical guidelines for assessing anxiety and depression (the GAD-2 and Whooley questions respectively) [9]. Initially 159 women enquired about the study, of whom 87 did not return the eligibility questionnaire or respond to follow up, and 31 did not meet inclusion criteria. The remaining 41 women were invited to interview. The final sample therefore consisted of 41 women (17 in Scotland and 24 in England). Women who participated were entered into a draw to win one of two £50 vouchers.

Sample characteristics are given in Table 1. The majority of the sample were White Caucasian and employed. Participants were pregnant (61%) or postpartum (39%), and 46% scored over the cut-off for probable depression and/or anxiety.

**Table 1:** Sample characteristics

	N (%)
Age (years)	
23	1 (2·4)
25–29	10 (24·4)
30–34	11 (26·8)
35–40	19 (46·3)
Recruitment Site	
England	24 (58·5)
Scotland	17 (41·5)
Ethnic Background	
White Caucasian	38 (92·7)
Asian	1 (2·4)
Multiple Ethnic Groups/Mixed	2 (4·8)
Education	
A-Level/Other Level 3 Qualification	6 (14·6)
Degree/Other Level 4 Qualification	11 (26·8)
Higher Degree/Level 5 + Qualification	24 (58·5)
Employment Status	
Employed	38 (92·7)
Unemployed	2 (4·8)
Other (Student)	1 (2·4)
Pregnancy/Postnatal Stage	
12 Weeks	6 (14·6)
22 Weeks	6 (14·6)
31 Weeks	13 (31·7)
6 Weeks Postpartum	16 (39·0)
Probable depression or anxiety	
Depression	17 (41·5)
Anxiety	7 (17·1)

### Measures

Five versions of four measures were assessed: the GAD-2, GAD-7 [10]; Whooley questions [11]; CORE-10 [14], and SAAS [16]. A description of each measure and the rationale for its inclusion is given in Table 2.

**Table 2:** Scale characteristics and rationale for inclusion

Questionnaire	Generalised Anxiety Disorder (GAD-2/7)	Whooley Questions	CORE-10	SAAS
Description	Two questions taken from the original 7-item version. If a woman scores 3 or above on the GAD-2, the GAD-7 can be used for further assessment and referral	Two questions widely used in maternity services to assess depression	A 10-item measure of psychological distress, which covers a range of symptoms of distress and associated functioning	A clinically derived 10-item measure that includes both general and pregnancy-specific anxiety symptoms
Items	How often during the previous two weeks have you been bothered by: 1. Feeling nervous, anxious or on edge 2. Not being able to stop or control worrying 3. Worrying too much about different things 4. Trouble relaxing 5. Being so restless that it is hard to sit still 6. Becoming easily annoyed or irritable 7. Feeling afraid as if something awful might happen	During the past month have you often been bothered by: 1. Feeling down, depressed or hopeless 2. Having little interest or pleasure in doing things	How often have you felt this way over the last week: 1. I have felt tense, anxious or nervous 2. I have felt I have someone to turn to for support when needed 3. I have felt able to cope when things go wrong 4. Talking to people has felt too much for me 5. I have felt panic or terror 6. I made plans to end my life 7. I have had difficulty getting to sleep or staying asleep 8. I have felt despairing or hopeless 9. I have felt unhappy 10. Unwanted images or memories have been distressing me	How often, in the past two weeks: 1. My anxiety stopped me from doing things 2. I felt panicky for no good reason 3. I felt unable to cope 4. I worried that something may be wrong with my baby 5. Thoughts got stuck in my head 6. I avoided people 7. I could not control my anxiety 8. I have had negative thoughts about childbirth 9. I did not feel worthy of being a mother 10. My worries overwhelmed me
Responses	not at all/several days/more than half the days/nearly every day	yes/no	not at all/only occasionally/ sometimes/often/most or all of the time	never/rarely/sometimes/often/ always
Rationale for Inclusion in Study	Acceptable validity and reliability of the GAD-7 have been reported in perinatal women. [21] The Royal College of Psychiatrists, NICE Guidelines and NHS England recommend the GAD-2/7 as one of a few measures to use with perinatal women. [8, 22] However, the current evidence for use of the GAD-2/7 with UK perinatal women is limited with some evidence the GAD-2 is inappropriate for screening for anxiety in pregnancy. [12]	High sensitivity and variable specificity in identifying perinatal depression has been reported. [23] Whooley Questions may also be appropriate to assess perinatal anxiety and other mental disorders. [11] Potential suitability to assess perinatal anxiety and mental disorders is based on its widespread clinical use and indication that it identifies other mental disorders. However, current evidence for the Whooley Questions to assess perinatal anxiety is limited.	Derived from the larger CORE-OM, a well-established measure used in counselling and clinical psychology services in the UK. [24] The CORE-10 is a preferred outcome measure for the Improving Access to Psychological Therapies services in England. [25] It is recommended as one of a few measures to use with perinatal women. [22] Good psychometric properties with perinatal women have been reported, but current evidence is limited. [13]	Developed from a systematic review of existing anxiety scales; interviews with women who experienced antenatal anxiety; and a Delphi study with clinicians with expertise in perinatal mental health. The SAAS has excellent sensitivity; good specificity; and showed superior performance compared to the GAD-2 and GAD-7. It was also considered acceptable to pregnant women. It includes both general and pregnancy-specific anxiety items. However, the evidence on the accuracy and acceptability of the SAAS is limited to one study. [15]

### Data collection

Women interested in participating were sent a participant information sheet, consent form, and brief eligibility questionnaire to obtain information on demographic characteristics, the Whooley Questions and GAD-2. All women indicating anxiety or depression on either of these measures were encouraged to talk to their midwife or GP and sent details of support organisations.

Interviews were conducted by researchers with training in cognitive interviewing methods (AS, LW, RC). The interview included a verbal introduction to perinatal mental health assessment and instructions on the cognitive interview process. Following that, a worked example of a question and response was conducted with the participant [26]. Two cognitive interviewing techniques were used. [21] First, the participant was asked to read each question in the four measures aloud and ‘think aloud’ as she came to an answer, enabling respondent-driven data. Second, probes were used to explore specific aspects of the cognitive model of survey question response developed by Tourangeau (see Table 3) [18]. Spontaneous probes were used to explore any hesitation, confusion or uncertainty. Participants were also asked about the acceptability and relevance of items to perinatal women. Questionnaires were counterbalanced to avoid order bias so each measure was presented to participants as first, second, third or forth a similar number of times. Interviews were conducted in person (*n* = 39) or by telephone/online (*n* = 2), between July 2019 and January 2020 and lasted approximately 60 min (range 34–95). Interviews were recorded, transcribed verbatim and transcripts de-identified.

**Table 3:** The four-stage cognitive model of survey response with examples of probes

Model stage	Explanation	Examples of probes
Comprehension	What does the respondent understand by the words and phrases in the question? What does the respondent believe the question is asking her?	What does ‘being able to cope’ mean to you? (CORE-10) What is the difference to you between feeling nervous, anxious, or on edge? (GAD-7)
Retrieval	How does the respondent access and retrieve information needed to answer the question? What strategies does she use? E.g. counting individual events, or using an estimation strategy?	How did you come to this answer? How are you deciding on how often you felt this way?
Judgement	What decisions is the respondent making about how to answer the question? E.g. how plausible, accurate, motivated, and socially desirable is their response?	Is there anything that would make you not want to answer this question? I noticed you hesitate – can you say a bit about why that is?
Response	How does the respondent match her internally generated answer to the response categories given in the questionnaire?	Is there a category there that you think matches what you feel? What do you think about these response options?

### Data analysis

Transcripts were imported into NVivo12 software [27]. Framework analysis was used to evaluate all items according to the dimensions of the Tourangeau model [18]. Additional coding categories were relevance and acceptability of individual items, response options, timeframe, and general comments.

Analysis was conducted by three researchers (AD, AS, LW) who coded transcripts from all participants, focussing on different measures. Transcripts were coded line by line with codes from the framework or new descriptive codes. Coding was discussed at regular meetings throughout the analysis phase to ensure reliability. To check interrater reliability another author (RC) checked 5% of quotes and interrater reliability was 82%. All disagreements were resolved by discussion. Categories were revisited to check there was sufficient evidence to support themes and a summary of findings was agreed by all authors. Standards for Reporting Qualitative Research [28] and Consolidated Criteria for Reporting Qualitative Studies (COREQ) guidelines [29] were adhered to.

To examine acceptability using Tourangeau’s model it was necessary to create a threshold above or below which items were classified as having positive or negative characteristics. For this we set a threshold of 20% or more participants (i.e. at least 8 of 41 participants) expressing difficulties or benefits for a particular issue to be considered notable. The rationale for this was that 20% (or 1 in 5) is a substantial enough proportion of the sample (and potentially the population) that problems should be considered and possibly addressed.

### Ethical approval

Informed consent was obtained from all participants. Ethical approval for all research protocols was obtained from the City, University of London School of Health Sciences Research Ethics Committee (ETH1819-0689).

## Results

Findings are presented on the acceptability and ease of use of each measure using quantitative data from the cognitive interviews, supported by qualitative data summarising women’s views (EP = English participants, SP = Scottish participants). Information on acceptability and relevance to perinatal women, and acceptability of response options is then reported.

### Acceptability of measures

All measures were acceptable to women and they were able to complete them easily. Table 4 shows the proportion of items in each measure viewed as positive or problematic by participants. The GAD-2 had the highest proportion of both positive and problematic components. Of the other measures, three had a reasonable number of items with positive comments: CORE-10, Whooley questions, and SAAS. In contrast, the GAD-7 received the fewest positive comments overall. The fewest problematic components were in the Whooley and SAAS.

**Table 4:** Proportion of Toureangeau’s components considered positive or problematic

	Components described positively % (n)	Components identified as problematic % (n)	Problems with comprehension Mean (SD)	Problems with retrieval Mean (SD)	Problems with judgement Mean (SD)	Problems with responding Mean (SD)	Mean problematic comments per component
GAD-2 (2 items, 8 components)	75% (6)	37% (3)	7·7 (0·5)	4·0 (0)	8·0 (2)	8·5 (1·5)	**28·2**
GAD-7 (7 items, 28 components)	18% (5)	21% (6)	6·6 (2·8)	5·7 (1·7)	6.1 (2·6)	4·4 (2·9)	**22·8**
Whooley (2 items, 8 components)	37% (3)	12% (1)	6·5 (1·5)	2·0 (0)	6·5 (0·5)	6·0 (1)	**21·0**
CORE-10 10 items, 40 components)	40% (16)	20% (8)	6·9 (3·7)	5·1 (1·7)	4·8 (2·3)	2·5 (2·0)	**19·3**
SAAS (10 items, 40 components)	35% (14)	15% (6)	6·4 (3·1)	5·2 (2·3)	1·9 (1·6)	2·0 (1·3)	**15·5**

NB: Each item was evaluated for four components (comprehension, retrieval, judgement, response), hence differing component numbers for each measure. Components were identified as problematic or positive if at least 8 women (20% of sample) raised this

Table 4 also gives the mean number of items described as problematic on Toureangeau’s components of comprehension, retrieval, judgement or responding. This shows the lowest mean number of problematic items were found in the SAAS and CORE-10. Thus, overall, the SAAS and CORE-10 had the most positive comments and least problematic issues. Conversely, the GAD-7 and GAD-2 had the least positive comments and most problematic issues.

### Qualitative information on different measures

The **GAD-2 and GAD-7** had the fewest positive comments and most problematic issues. In terms of problematic items: *‘Feeling nervous, anxious or on edge’* was reported as problematic for comprehension by 20% of participants due to the item combining three different issues (nervous, anxiety, on edge). Conversely, 27% of women thought the concept of being *‘on edge'* was easy to understand.

*I think the addition of ‘on edge’ really helps, because it could just be like a fleeting, oh I’m a little bit, you’re not actually nervous, but you’re almost on edge about being nervous and that’s a different thing. (EP05)*

The item ‘*Worrying too much about different things*’ was reported as difficult to comprehend by 27% of participants because it was too similar to another item *‘Not being able to stop or control worrying’.* Participants found it difficult distinguishing between a normal level of worry and excessive worrying, especially in the context of pregnancy or having a young infant.

*So I think if you said ‘Worrying about different things?’, I’d probably go “Yes, and I should do”, it’s almost that is where I’m at, that I need to be thinking about all the stuff that has to happen and different bits that we need to think about; whereas ‘worrying too much’, I think that’s why I scored it lower, because actually I would say it’s a reasonable amount of worry. (EP12)*

Similarly, 22% of participants thought the item *‘Becoming easily annoyed or irritable’* was problematic in terms of retrieval because of uncertainty over whether this symptom was due to anxiety or normal perinatal issues such as tiredness.

*I always answer nearly every day because I do feel annoyed pretty much every day at something, … anxiety-related or not. (EP01)*

Items in the GAD-2 and GAD-7 that were evaluated positively included *‘Not being able to stop or control worrying’* which was viewed as easy to comprehend and respond to by 24% of participants; and *‘Being so restless that it is hard to sit still’* which was viewed as easy to respond to by 31% of participants.

The **CORE-10** was one of the measures with the most positive comments and least problematic items. Only one item on the CORE-10 was viewed as problematic without counter-balancing positive views. This was ‘*I have felt unhappy*’ which was viewed as difficult to comprehend and judge due to being unsure of what being *‘unhappy’* meant (22% of participants); and problems differentiating between unhappiness and worry (24%).

*“When I think about the word unhappy, the more I think, oh I don’t know if I was actually unhappy at that moment, or whether I was just expressing an emotion of worry”* (EP06)

Five items on the CORE-10 attracted mixed views and were viewed as both positive and problematic by different participants. The item ‘*I have felt able to cope when things go wrong*’ was viewed positively by 22% of participants who thought the concept of ‘to cope’ was easy to understand, and 32% of participants who thought it was easy to retrieve their answer because they could remember times when they did not cope well.

*I’d say often because when she was … she was quite sick yesterday. I took her to the doctor. At that point I was like, oh, I don’t have the facility to cope with that myself in a physical way. (SP02)*

However, another 22% of participants found this item hard to comprehend, querying what *‘wrong’* meant and the magnitude of this term.

*Does it have to be a big thing? Small thing? It’s tricky cos I’m not sure what I would count as going wrong. (EP01)*

Similarly, the item ‘*Talking to people has felt too much for me*’ was viewed as clear and straightforward (27%) and easy to comprehend (20%).

*It’s that sort of feeling of being overwhelmed that actually you need to shut yourself off or come away from people, because of how you’re feeling emotionally. (EP12)*

but was problematic for some participants who thought the question was too broad.

*It hasn’t felt too much for me to talk to friends and family, but sometimes when strangers come up to you and comment really nice things about your baby, but baby’s crying and you just want to feed her, then that’s felt too much for me. (EP02)*

The item *‘I have felt panic or terror’* was thought to be easy to comprehend (41%) and respond to (39%) due to the words ‘panic’ and ‘terror’ having clear meanings.

*Again, this is very easy, because panic and terror are really strong words. (EP17)*

However, a smaller proportion of participants (22%) found it difficult to comprehend because it includes two emotions and the word *‘*terror’ was viewed as extreme.

*If it was just ‘I've felt panic’ I would probably say [yes], [but] because it's… next to the word terror I'm looking at that and saying no I've not been terrified. (EP15)*

The item ‘*I have felt despairing or helpless’* was evaluated as easy to comprehend (27%) and respond to (20%) by some participants because the strength of emotions in ‘*despairing or helpless*’ was clear.

*I mean I think that’s quite clear on what kind of emotions, I mean I interpret that as feeling very heightened emotions, where you felt like you couldn’t ask for help from anyone. (EP02)*

However, others found it harder to understand and respond to (20%) due to uncertainty over the meaning of the word ‘*despairing’*.

*In a way, I don’t really know what to make of it. So I’d probably say only occasionally because I wouldn’t want to put something too serious on something that I don’t really get. (SP08)*

Finally, the item ‘*Unwanted images or memories have been distressing me*’ was difficult to comprehend for 22% of participants as they were unclear what kind of images the item referred to. It was therefore difficult for them to retrieve their answers because they were unclear whether the question was relating to flashbacks or overthinking.

*I don’t know whether this question wants to know whether I experience flashbacks or whether I’m ruminating and bringing on these unwanted images. (EP01)*

However, another 20% of participants said they found it easy to retrieve their answers and match their experience to the response options.

*Yeah, you’re walking down the stairs and you can see yourself fall and crush the baby, and you’re like, oh. You’re walking down the street and, you know, like you see somebody walking and you think what if they steal the baby, and you can visualise it and stuff…so that would be sometimes. (SP02)*

The **SAAS** was the other measure with the most positive comments and least problematic items. No items on the SAAS were viewed as problematic without counter-balancing positive views. Four items were only viewed positively: *‘my anxiety stopped me from doing things’* was viewed as easy to comprehend (27%), judge (22%), and respond to (27%); the item *‘I felt panicky for no good reason’* was viewed as easy to comprehend (39%); the item *‘I avoided people’* was reported as easy to comprehend (22%); and the item *‘I did not feel worthy of being a mother’* was viewed as easy to respond to (20%).

Five items on the SAAS had mixed views, with participants evaluating them positively or as problematic. Similar to the CORE-10, the item ‘*I felt unable to cope'* was viewed as easy to comprehend (22%) and respond to (20%) by participants who could relate to the term ‘*coping’*.

*I mean I guess to me, ‘coping’ is, again, you’re able to take care of everybody’s normal needs. So if you weren’t able to do that that’s you not coping. (SP06)*

Other participants (22%) found it hard to comprehend because the term ‘*coping’* was harder for them to define.

*‘Unable to cope’, I don’t know, it’s … not very clear to me, so I would say probably never, because …even if I am anxious, then after a while it just goes, I try to manage it, even if I can’t, it just goes, so it’s not that terror, unable to cope, but it’s I don’t know it’s kind of, it’s not straightforward this question. (SP04)*

The item ‘*I worried that something may be wrong with my baby*’ was viewed as problematic for retrieval (22%) because it is common in pregnancy and as a new mum to worry, but there was uncertainty over whether this was normal or should be a concern.

*It’s just a constant level of actually worry and anxiety and maybe it’s as a first-time mum not knowing, … what’s normal, what’s not and is there a normal? … I’m constantly checking his temperature and wondering do I need to go to the GP. (EP15)*

However, the same item was viewed positively for judgement (22%) by participants who were able to differentiate between normal worries about the baby and real concern.

*I think when they're this small then you are, anxious isn't the correct word, yeah you're just concerned I guess. But I wouldn't have specifically said there was something wrong with him. So ‘never’ I guess I would answer for that one. (SP01)*

The item ‘*Thoughts got stuck in my head ‘* was evaluated as difficult to comprehend (29%) and retrieve (20%) because of uncertainty over the type of thoughts this referred to.

*I don’t think it is a clear question because it makes me think, okay, are you talking about intrusive thoughts or talking about just having negative thoughts? (EP17)*

However, a similar proportion of participants evaluated this item as easy to comprehend (27%) and respond to (22%) because they were able to separate periods of negative thoughts from ongoing rumination.

*To me that means negative thoughts getting stuck in your head so it’s whatever your particular worry or concern is. Where you able to process it and move past it? (SP06)*

The item ‘*I could not control my anxiety*’ was viewed by some participants as difficult to comprehend (24%) due to difficulties interpreting the meaning of ‘control’.

*I think that’s difficult to interpret that. Would it be a case of, I mean when I wake up and I feel angsty or stuff like that it just happens and that’s a thing I've learnt also with depression, is just sometimes you need to go with the flow and just accept you’ve got a bad day and just ride the wave. (EP03)*

Conversely, other participants found it easy to comprehend (24%) and respond to (22%) as it implicitly acknowledges that anxiety can be normal but becomes problematic when anxious thoughts take over.

*Feeling anxious, yes, but controlling it I would say never because I feel that I do have a handle on it and I can, you know, cope with it, put it to bed. (SP14)*

Finally, the item *‘My worries overwhelmed me’* was difficult for some participants to comprehend (20%) due to uncertainty of whether the item referred to overwhelming worries, or worrying in general.

*It’s so tricky … is it asking how often my worries overwhelmed me, or is asking when I was worried did I feel overwhelmed, … is it a quantity question kind of thing? How often have I had overwhelming worries when I’ve been worrying, or in general. (EP01)*

However, participants found it easy for judgement (27%) because they could easily assess the times they had been overwhelmed.

*I would put ‘sometimes’ because when I have worries I sometimes feel overwhelmed but other times I don’t. I guess I can kind of overcome them, or distract myself, or that kind of thing. (EP01)*

The **Whooley** only consists of two questions. The item *‘During the past month have you often been bothered by having little interest or pleasure in doing things’* was viewed positively as easy to comprehend by 20% of participants. The item ‘*During the past month, have you often been bothered by feeling down, depressed or hopeless?*’ was viewed positively for comprehension (29%) and judgement (22%) by participants who found it easy to comprehend that being ‘*bothered’* meant having a negative impact on life.

*So feeling helpless and feeling down sometimes as we kind of talked through but I haven't been bothered by it, I've kind of accepted that as you know part of being a mother and part of the new experience. (EP09)*

However, others (20%) found it difficult to comprehend because it was wordy and terms were unclear.

*The difficulty probably with this question is that you could interpret those three bits quite differently. So feeling down, I would say yes, but also … that’s potentially quite normal, whereas depressed or hopeless, I feel a bit stronger, so probably less identify with those bits. (EP12)*

### Acceptability of measures and their relevance to pregnancy and postpartum

Items were also evaluated for relevance and acceptability to participants during pregnancy or after birth. Results indicated the GAD-7 had the most items not considered relevant by participants: ‘*Becoming easily annoyed or irritable’* (24%) and ‘*Trouble relaxing’* (32%). This was largely due to being unable to separate whether these symptoms were due to being pregnant or being the parent of a young baby, or due to mental health problems.

*I wasn’t having trouble switching off from the things that were making me anxious, so I was able to enjoy family time, if that’s what ‘relaxation’ is for me, at the moment. It’s certainly not sat by a pool reading a book [chuckling]… I guess it’s not, just not really relevant at the moment. EP06*

The CORE-10 item ‘*I have had difficulty getting to sleep or staying asleep’* was also not considered relevant (46%) due to sleep being commonly disturbed in pregnancy and after birth, due to discomfort or caring for a newborn.

*I felt again [it] was like, a genuinely insulting question to ask someone with a six week old baby [chuckling]. EP19*

The Whooley item ‘*during the past month have you often been bothered by having little interest or pleasure in doing things?’* was considered problematic in relation to the perinatal period by a minority (12%) because during pregnancy tiredness and sickness are common and can be limiting, and after birth women are focusing on baby care rather than doing things they would get interest or pleasure from.

*Well what sort of things are you expecting a new mum to do, because a lot of what they’re doing is very mundane and around the baby. It’s not like they’re doing something that is … you know they’re not going out with their friends that you would expect to pleasure-inducing, like, changing a baby’s nappy ten times a day isn’t expected to be pleasurable. It’s just … these are the realities of a newborn that you have to do. SP06*

Finally, participants were asked if any of the items were unacceptable to them. This highlighted potential issues with two items: the CORE-10 item ‘*I made plans to end my life*’ was viewed as unacceptable by 27% of participants because of the extreme nature of the question and uncertainty over whether people would answer truthfully.

*I think it's a daunting question. I know that it's a standard regardless of like in a mental health situation we need to ask those questions. It's a pretty scary question to ask and I think there's also a limit, planning to end my life or would the world be better without me or would things be better if I wasn’t there. You know there's different levels of it as well. I find that question scary and every time I get asked that I'm like ‘I understand why you need to ask it but it's extreme’. EP03*

However, more participants (49%) viewed this item positively due to the importance of knowing if a person is actively suicidal.

*Yes, I think it’s a good one to ask and I like the way that that’s worded, compared to some of the other questions you get, which is just like, because that’s, because I think a lot of people have thoughts of not wanting to be here, but that’s different to actually having made a plan and I think it’s good that it specifically asks that. SP09*

The SAAS item ‘*I did not feel worthy of being a mother’* was viewed as unacceptable by 22% of participants because they found the word ‘*worthy’* to be judgemental and harsh, especially for pregnant women who did not see themselves as a mother yet.

*Number nine, ‘I did not feel worthy of being a mother?’, well I’m not a mother yet, and I’m quite aware that I won’t be a mother until September, so I don’t feel like a mother. SP16*

### Evaluation of response options

Overall, participants preferred non-binary response options. The binary *‘Yes/No’* response options on the Whooley were viewed as too limited by some (32%).

*‘Yes’ or ‘no’? It’s a stark choice isn’t it, you’ve got to decide… decide your feelings and strike a choice and I guess, I mean there’s a way in which, it’s like writing down “Are you alright?”, and so it’s asking you to put all of the rest of that stuff, in a single judgement and be like, “You alright love?” “Yes or no?”.” EP19*

Non-binary response options were preferred, although some participants struggled to interpret differences between the different response options e.g. *‘only occasionally’* and *‘sometimes’* (CORE-10; 27%); *‘sometimes’* and *‘often’* (SAAS; 15%); *‘not at all’* and *‘several days’* (GAD-2 & GAD-7; 73%):

*…several days. How many does that equal to me? That would be … equals about five days. More than half the days equals about eight days. Nearly every day equals probably about 12-14 days. So I guess it’s quite … yeah, I guess it’s more of a jump I guess from between several days to more than half the days. Several days could be like three or four, I guess. More than half the days has got to be at least eight, which is like double. Yeah. So maybe there needs to be something in the middle.” EP01*

### Evaluation of timeframes

The timeframes used ranged from *‘the last week’* (CORE-10), *‘two weeks’* (GAD, SAAS) to *‘one month’* (Whooley). Participants varied in which timeframe they preferred, with positive and negative views on all options. The one-week timeframe had mixed reviews as too short (46%) but easy to remember (10%). The two-week timeframe was viewed positively by some participants (41%) but others preferred a shorter or longer timeframe (12%).

*I think [in] pregnancy I'm not sure it matters too much, but I think postnatal each week is so different, so if you asked me in my third week when I was crying all the time from my hormones that would be very different between week three and four. So maybe seven days, because [the baby] changes so often as well… It's tricky yes, I think there's too much change for fourteen days, I think it's quite long. SP03*

The one-month timeframe also had mixed views as difficult to recall feelings compared with shorter timeframes (27%) but more likely to be clinically meaningful (15%).

*It says month doesn’t it, I’d say that probably is more appropriate, I think it maybe gives you a chance… to flag something up that allows a conversation to probe further, then you’re giving yourself more opportunity for that. EP06*

## Discussion

This study examined the acceptability of five versions of four measures to assess perinatal anxiety to perinatal women. Overall, the measures evaluated were considered acceptable and relevant by participants, but items varied in whether they were viewed as positive or problematic in terms of comprehension, judgement, retrieval and responding. Overall, the SAAS and CORE-10 had the lowest mean number of problematic components. The Whooley questions also performed well. The GAD-2 and GAD-7 had the greatest number of problematic components and, notably, the GAD-7 was also the measure with most items considered not relevant to perinatal women. This poorer performance of the GAD is concerning given it is currently the recommended screening tool for perinatal anxiety in the UK [8]. Recent studies also found the GAD-7 had poor diagnostic accuracy with perinatal women [13, 30].

Results for the Whooley questions were mixed. The Whooley is the recommended screening tool for perinatal depression in the UK and has high sensitivity and specificity for identifying perinatal depression [24]. In this study the Whooley performed well and had least problems for ease of retrieval, although some participants questioned the relevance of one of its questions to the perinatal period. The binary *‘Yes/No’* responses were also thought to be too limiting by some participants.

The SAAS was the least problematic measure in relation to comprehension, judgement and responding; and had a good level of positive comments. The SAAS includes both general and pregnancy-specific anxiety items, so differs from the other measures in this respect. Original testing of the measure showed it had good sensitivity, specificity and performance compared to the GAD-2 and GAD-7. [16] However, some participants struggled to interpret the response options and the item *‘I did not feel worthy of being a mother’* was not considered acceptable by some participants.

The CORE-10 had a good level of positive comments. The CORE-10 was derived from the larger CORE-OM, a well-established measure used in psychology services in the UK [25]. However, the relevance of the item *“I have had difficulty getting to sleep or staying asleep”* to perinatal women was questioned. Some participants also struggled to interpret the response options. The item “*I have made plans to end my life*” was not considered acceptable by a quarter of participants despite being commented on positively by half of participants.

This research has a number of implications for healthcare practice and policy. First, findings highlight the variation between participants in the perceived acceptability of items, ease of use, and preferences in relation to response options and timeframe. It is perhaps unsurprising that what is acceptable and easy to use for one person is not the case for another. There was also no precedent for the appropriate threshold to use to identify ‘positive’ or ‘problematic’ components so our threshold of 20% or more of the sample was decided on the basis that 20% is a large enough proportion of the sample or population that it needs examining further.

Second, findings suggest the measures recommended for screening and assessment of perinatal anxiety in the UK, the GAD-2 and GAD-7, may not be the most acceptable or easy to use for perinatal women. This research suggests the SAAS and CORE-10 might offer more acceptable alternatives to assess perinatal anxiety/mental health with perinatal women. However, both might benefit from rewording of a few items to improve comprehension. Also, despite performing well, the SAAS and CORE-10 had the only items viewed as not acceptable by more than 1 in 5 women (feeling not worthy as a mother/making plans to end their life). Both these items ask about negative or extreme emotional states, so it is understandable they evoked strong views in some women. This raises the dilemma of whether measures with an ‘unacceptable’ question to more than 1 in 5 women should be used in clinical practice; or whether other priorities need to be considered, such as suicide being a major cause of maternal mortality [31]. In addition, a larger proportion of the sample commented positively on the suicide item, saying they understood the value of this question even if it was only applicable to a few women. Research is therefore needed to explore the assessment of suicidal ideation/intent in more detail.

Finally, it is important to recognise that self-report measures are not the only approach to screening or assessment of perinatal mental health. These measures are embedded in healthcare services where care relies on interpersonal interactions and relationships. Barriers, such as stigma and fear of consequences, may determine whether women are prepared to disclose difficulties [32]. There are important facilitators to this, such as a trusting relationship between the woman and healthcare professional and continuity of carer [32]. However, self-report measures do have advantages: they can be administered to large numbers of women at relatively low cost; they are quick and provide standardised assessment; factors which are likely to contribute to their inclusion in clinical guidelines [9].However, more research is needed on how to use self-report measures feasibly and effectively in the context of pressures on healthcare professionals and services, as well as the difficulty of onward referrals if treatment services are not available.

### Strengths and limitations

This is the first study to use cognitive interviewing to evaluate ease of use, relevance and acceptability of measures used to assess perinatal anxiety. Study limitations include the relatively small sample size and that the majority of the sample were highly educated, employed, and White Caucasian. The sampling strategy meant there was a high prevalence of self-reported depression and anxiety in our sample compared to the perinatal population. It is therefore important that future research looks at the acceptability and ease of use of these measures in population-based samples, as well as diverse groups. Measures were chosen based on clinical utility and the current evidence base on perinatal anxiety measures, but feasibility of conducting the research and the need to keep participant burden low meant that other potentially appropriate measures were not tested.

## Conclusions

The perinatal period provides important opportunities to identify and support women with anxiety and poor mental health. A brief self-report measure that is clear, relevant, and acceptable to women is important to identify those who are likely to benefit from support and intervention. This study found that all measures were acceptable but the SAAS and CORE-10 performed better than other measures. The GAD was the least acceptable measure, so clinical guidelines and services should consider replacing it. This study provides information on measures that are more acceptable, which should be used in conjunction with research on effectiveness and diagnostic accuracy to inform which measures are used for perinatal anxiety assessment in different settings. Information is also provided on items within each measure that might benefit from further clarification or development.

## Data Availability

Individual, participant-level data is not available but the corresponding author can provide sample-level data and information on request after publication. The study protocol is available at https://njl-admin.nihr.ac.uk/document/download/2034506.
